# The Role of TRP Channels in Nicotinic Provoked Pain and Irritation from the Oral Cavity and Throat: Translating Animal Data to Humans

**DOI:** 10.1093/ntr/ntac054

**Published:** 2022-02-24

**Authors:** Lars Arendt-Nielsen, Earl Carstens, Gordon Proctor, Yves Boucher, Pere Clavé, Kent Albin Nielsen, Thomas A Nielsen, Peter W Reeh

**Affiliations:** Center for Neuroplasticity and Pain (CNAP), SMI, Department of Health Science and Technology, School of Medicine, Aalborg University, Aalborg, Denmark; Department of Medical Gastroenterology, Mech-Sense, Aalborg University Hospital, Aalborg, Denmark; Neurobiology, Physiology and Behavior, University of California, Davis; Centre for Host-Microbiome Interactions, Professor of Salivary Biology, King´s College London, UK; Laboratory of Orofacial Neurobiology, Paris Diderot University, Paris, France; Centro de Investigación Biomédica en Red de Enfermedades Hepáticas y Digestivas (Ciberehd), Hospital de Mataró, Universitat Autònoma de Barcelona, Mataró, Barcelona, Spain; Nicotine Science Center, Fertin Pharma A/S, Vejle, Denmark; Mech-Sense & Centre for Pancreatic Diseases, Department of Gastroenterology & Hepatology, Clinical Institute, Aalborg University Hospital, Aalborg, Denmark; Institute Physiology and Pathophysiology, Friedrich-Alexander-Universität Erlangen-Nürnberg, Erlangen, Germany

## Abstract

Tobacco smoking-related diseases are estimated to kill more than 8 million people/year and most smokers are willing to stop smoking. The pharmacological approach to aid smoking cessation comprises nicotine replacement therapy (NRT) and inhibitors of the nicotinic acetylcholine receptor, which is activated by nicotine. Common side effects of oral NRT products include hiccoughs, gastrointestinal disturbances and, most notably, irritation, burning and pain in the mouth and throat, which are the most common reasons for premature discontinuation of NRT and termination of cessation efforts. Attempts to reduce the unwanted sensory side effects are warranted, and research discovering the most optimal masking procedures is urgently needed. This requires a firm mechanistic understanding of the neurobiology behind the activation of sensory nerves and their receptors by nicotine. The sensory nerves in the oral cavity and throat express the so-called transient receptor potential (TRP) channels, which are responsible for mediating the nicotine-evoked irritation, burning and pain sensations. Targeting the TRP channels is one way to modulate the unwanted sensory side effects. A variety of natural (Generally Recognized As Safe [GRAS]) compounds interact with the TRP channels, thus making them interesting candidates as safe additives to oral NRT products. The present narrative review will discuss (1) current evidence on how nicotine contributes to irritation, burning and pain in the oral cavity and throat, and (2) options to modulate these unwanted side-effects with the purpose of increasing adherence to NRT. Nicotine provokes irritation, burning and pain in the oral cavity and throat. Managing these side effects will ensure better compliance to oral NRT products and hence increase the success of smoking cessation. A specific class of sensory receptors (TRP channels) are involved in mediating nicotine’s sensory side effects, making them to potential treatment targets. Many natural (Generally Recognized As Safe [GRAS]) compounds are potentially beneficial modulators of TRP channels.

## Introduction

Tobacco smoking-related diseases, particularly cardiovascular diseases, cancers and chronic respiratory diseases, remain highly prevalent, and WHO has estimated that smoking kills more than 8 million people each year. This clearly demonstrates that—despite more than 50 years of anti-tobacco efforts and actions—smoking remains a leading but preventable risk factor.^1^ Although the majority of smokers are willing to stop smoking,^2^ quitting tobacco use is challenging. The main pharmacological treatments include nicotine replacement therapy (NRT), nicotinic acetylcholine receptor (nAChR) partial agonists such as varenicline and cytosine, and the antidepressant bupropion.^3^

NRT is an effective first-line medication to ease the transition from cigarette smoking to tobacco abstinence.^3^ Based on high-quality evidence from studies involving 64 640 participants, all licensed forms of NRT increased the rate of quitting by 50%–60%—regardless of type, dose and treatment setting.^4^ NRT products are developed for absorption of nicotine through the oral or nasal mucosa, as well as transdermal patches.^3,4^ Common side effects associated with oral NRT products include hiccoughs, gastrointestinal disturbances and, most notably, irritation, burning and pain in the mouth and throat.^4^ Discontinuation rates within the first treatment week amounted to 20% for NRT compared to only 2.3% for prescription-only medications including varenicline and bupropion,^5^ indicating that the local side effects may reduce adherence to NRT.

Hence, attempts are made to mask these unwanted side effects of oral NRT products. As summarized below, a vast amount of evidence exists that natural compounds can target transient receptor potential (TRP) channels, for example, from the monoterpene class including menthol, can modify the oral irritation and pain elicited by nicotine. The present narrative review will discuss current evidence on how nicotine contributes to irritation and pain in the oral cavity and propose strategies for modulating these bothersome side effects, thereby increasing adherence to NRT.

## Histology of the Epithelium and Mucosal and Submucosal Sensory Nerve Fibers in the Human Oropharynx

The oral and pharyngeal mucosal/submucosal structures belong to the anterior part of the digestive system and have an important role in ingestive behaviors, detecting mechanical and chemical properties of food, regulating mastication and swallowing, inducing salivation, etc.^6^ The oral cavity, the oropharynx, and the hypopharynx are covered by three types of mucosa according to their function,^7^ while the structures of the larynx are covered by respiratory mucosa.

Beneath the epithelia of the oral mucosa are found the multilayered lamina propria mainly containing fibroblasts and immunocytes, and the submucosa containing fibro-collagenous and elastic tissue and according to the location, adipose tissue, minor salivary glands, lymphoid tissue, and muscles cells. Besides the keratinocytes found in these different types of mucosa, other cell types including melanocytes, Langerhans and Merkel cells are found, the latter associated with nerve cells involved in tactile information. In the deeper layers of the connective tissue, the biggest nerve fibers and vascular vessels are found. The smaller ones originate respectively from capillary loops and axons innervating the papillary elements of the mucosa, reaching the basal lamina or trespassing it.

The sensory innervation of the oropharynx is complex, integrating exteroceptive (tactile and proprioceptive) and homeostatic stimuli (pain, taste, temperature, chemesthesis, etc.)^8^ through Merkel’s disks, Meissner’s corpuscles, Pacinian corpuscles, and free nerve endings, respectively.^9^ Many receptor subtypes, including Piezzo, TRP, ASIC, ENa, and AchR, are responsible for the transduction mechanisms (see chapter below).^10^ Taste receptors responding to salty, sweet, sour, bitter, umami, and fat compounds are mainly located within taste buds (TB) receiving specific innervation in the dorsum of the tongue and the soft palate, but also in the larynx, pharynx, and upper esophagus mucosas.

Lingual Papillae are complex structures containing TB, innervated by different nerves. For example, TB in fungiform papillae receive both perigemmal (outside of the TB) trigeminal innervation and intragemmal chorda tympani innervation, mainly intragemmal (see next chapter). Chorda tympani as well as trigeminal afferents are able to encode mechanical thermal and chemical stimuli but sensory interactions also occur between these nerves at the mucosal level, favored by the specific anatomical organization of the fungiform papillae.^11^ For example, stimulating trigeminal afferents decreases taste responses in chorda tympani and increases tongue temperature^12,13^

## Oral Irritation and Pain Elicited by Nicotine

Nicotine, contained in cigarette smoke and NRT products, elicits oral irritation and pain by activating receptors located on the sensory nerve endings in the oral cavity and throat.^14^

**Figure 1.**
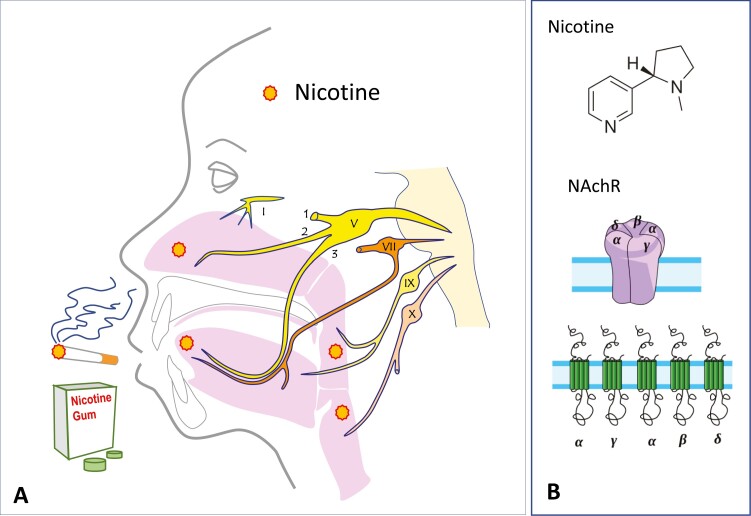
(A) Nicotine present in cigarettes and therapeutic substitutes evokes oral, nasal, and throat irritation through activation of several receptors distributed in the oro-naso-pharyngeal mucosas (see Figure 2). The structure of these mucosa is different as well as their innervation, provided by different cranial nerves (V, VII, IX, X) (see text for details). Nicotine acts also on several brain structures responsible of the craving effect. I = olfactory nerve; V = trigeminal nerve; 1: ophthalmic subdivision; 2: maxillary subdivision; 3: mandibulary subdivision; VII = facial nerve; IX = glossopharyngeal nerve, X = vagus nerve. (B) N**i**cotine (up, molecular structure) acts on neuronal nicotinic acetylcholine receptors (nAchR) (medium) made up of seven subunits (bottom) (see text for details). (Images from the right panel are used under Creative common license File:Ach rezeptor nicotinisch.png) (https://commons.wikimedia.org/wiki/File:Ach_rezeptor_nicotinisch.png?uselang=fr).

The pain sensation results from the activation of nociceptive C-fibers, for example, in the oral or nasal mucosa^15^ and lungs,^16^ eventually leading to neuronal excitation in the brainstem (Figure 1).^17^ The trigeminal nerves innervating the oral cavity supply afferent information on temperature, touch and pain and directly project to second-order neurons in the main trigeminal nucleus and the spinal trigeminal nucleus.

## Anatomy of the Human Sensory Oropharyngeal Pathways Related to Swallowing: First Site of Interaction with Nicotine

The sensory innervation of the oropharynx and the larynx involves five of the cranial nerves (Figure 1). The hypopharynx and the larynx are innervated by the internal superior laryngeal nerve of cranial nerve X. The sensory inputs are transmitted through all these nerves to the central pattern generator located in the medulla oblongata of the brainstem and to the somatosensory cortex and subcortical structures.^9^

Despite this overview, our knowledge on the sensory innervation and the receptors expressed in the human oropharynx and larynx is still limited. Most information on this topic comes from studies exploring the physiology of the swallowing function, the pathophysiology of oropharyngeal dysphagia (OD), and the basis for OD treatments based on oropharyngeal sensory stimulation as a therapeutic strategy for patients with swallowing disorders.

Oropharyngeal and laryngeal sensory innervation account for the fine perception required for proper coordination of the swallow response with airway protection mechanisms to avoid aspirations during swallowing. It is well known that the oropharyngeal mucosa is sensitive to a wide range of sensory stimuli through specialized structures and free nerve endings, and our studies found TRPV1, TRPA1, and TRPM8 widely expressed in all these sensory elements in the human oropharynx and larynx.^18,19^ Clinical studies clearly described that patients with OD presented a severe impairment of pharyngeal sensory function as a key pathophysiological element for swallowing dysfunction. In addition, pharmacological studies in humans found that oral administration of TRPV1, TRPA1, and TRPM8 agonists increased spontaneous swallowing frequency and improved the biomechanics and the neurophysiology of the swallow response in dysphagic patients. All these results pave the way for a pharmacological strategy targeting TRP receptors to develop a drug for patients with OD, the same pathway as used by nicotine, NRT and GRAS compounds described in this review. Progress in this area will contribute to clinical improvements in both sides and the development of effective drugs for swallowing disorders and smoking cessation.

## Pharmacology of Neuronal Nicotinic Acetylcholine Receptors (nAChRs) and Transient Receptor Potential (TRP) Channels

Prolonged systemic administration of nicotine was shown to sensitize trigeminal nociceptive neurons by increasing the expression of several signal transduction proteins such as mitogen-activated protein kinase and protein kinase A and of pro-inflammatory cytokines such as tumor necrosis factor alpha in trigeminal ganglia (TGs) (= peripheral sensitization) and the spinal trigeminal nucleus (= central sensitization).

In numerous electrophysiological and calcium imaging studies conducted in rodents, high micromolar concentrations of nicotine excited part of the neurons from ganglia involved in nociception, that is, in TGs, dorsal root ganglia (DRGs), and nodose/jugular ganglia.^16,20^

These studies revealed that nicotine’s irritant effect is mainly mediated by the activation of neuronal nAChRs located in these nociceptive nerve endings. In contrast to the G protein-coupled muscarinic receptors, neuronal nAChRs belong to the superfamily of Cys-loop ligand-gated ion channels. Only alpha7 subunits heterologously expressed show some G protein-binding ability that prolongs the nicotine-stimulated intracellular calcium rise after channel closure.^21^ They are composed of five nAChR subunits surrounding a cation (Na^+^, K^+^, Ca^2+^)-permeable channel pore. Nine α subunits (α2–α10) and three β subunits (β2–β4) are known that can be assembled to either homopentameric (α7, α9) or heteropentameric (α2–α6 with β2–β4) receptors with specific functional and pharmacological properties. Whereas the most abundant nAChR subtypes in the brain are α4β2-containing receptors and α7 receptors, α3β4-containing receptors represent the most widely expressed nAChR subtypes in the peripheral nervous system^22^ in human DRGs α3β2.^23^ Inhibition of nicotine’s action in rat nociceptive neurons by mecamylamine and dihydro-beta-erythroidine suggested the involvement of α3β4 and α4β2-containing nAChRs. Involvement of homomeric α7 nAChRs was demonstrated by methyllycaconitine (MLA) antagonism. Also, α6β4-containing nAChRs seem to play a role in nociceptive neurons.^24^ However, the species differences in nAChR subunit composition and thus in nicotine-evoked signals in sensory neurons should not be neglected: In an electrophysiological study investigating human DRGs, nicotine elicited robust, slowly activating and deactivating currents in 85% of neurons that could not be blocked by mecamylamine but by the α7-specific antagonist MLA. In contrast, in mice, most nicotine-induced currents appear transient and sensitive to MLA blockade, and in rats, both, mouse-like transient and slow currents were detectable.^23^ In the mouse trachea, MLA was completely ineffective, in contrast to mecamylamine in low micromolar concentration.^20^ This highlights the differential nAChR subunit composition in different species.^23^

However, focusing on peptidergic nerve endings in the mouse trachea, releasing CGRP upon stimulation, MLA was completely ineffective in contrast to mecamylamine in low µM concentration, which indicates predominance of heteropentameric neuronal nAChRs like in humans.^20^

Moreover, ACh acts on non-neuronal cells, largely through homopentameric nAChR expressed, for example, by immunocytes that are involved in chronic orofacial pain and irritation conditions.^25^ Other cells like solitary chemosensory cells coupled with trigeminal fibers expressing nAChRs might also play a role in oropharyngeal irritation.

In addition, recent evidence suggests the involvement of TRP channels in the local irritation and pain induced by nicotine. In an electrophysiological study, micromolar nicotine concentrations activated heterologously expressed mouse and human TRPA1 receptors, as well as native TRPA1 in mouse TGs. The nicotine effect was not abolished by the nAChR blocker hexamethonium and was strongly reduced in TRPA1-deficient mice, indicating that this effect was independent from nicotine’s agonistic action at nAChRs. Interestingly, TRPA1 activation could be blocked by millimolar concentrations of the nAChR antagonist mecamylamine that has structural similarity to the known TRPA1 inhibitor camphor. Thus, mecamylamine cannot be regarded as a selective nAChR antagonist, at least when using the compound at high concentrations. In the same study, TRPA1 was demonstrated to be involved in the nicotine-induced airway constriction reflex in mice, further highlighting the potential relevance of nicotine’s interaction with TRPA1.^26^

Nicotine can also modulate the capsaicin receptor TRPV1 as1 mM nicotine increased the capsaicin-activated currents in rat TG neurons and in Chinese hamster ovary (CHO) cells heterologously expressing TRPV1. This sensitizing effect was not mediated by nAChRs as it was unchanged in the presence of the nAChR antagonist mecamylamine and was also evident in CHO cells in which nicotine did not activate currents.^27^ Another Ca^2+^ imaging study in rat DRGs showed a desensitization of TRPV1 channels following nicotine-induced activation of nAChRs.^28^

In studies measuring the nicotine-stimulated release of calcitonin gene-related peptide (CGRP) from the isolated mouse trachea, a bimodal, inverted U-shaped concentration-response relationship involving both nAChRs and TRP channels was established for nicotine: the curve demonstrated a first maximum at a nicotine concentration of 100 µM with a half-maximal effective concentration (EC_50_) of ~30 µM, a nadir between 0.5 mM and 10 mM, followed by a second moderate increase at 20 mM. The nicotine effect in the micromolar range was solely mediated by the activation of nAChRs, most likely the α3β4 subtype. In contrast, the effect induced by millimolar nicotine concentrations also involved TRPA1 and TRPV1 channels. Thus, nAChRs located on mouse tracheal sensory nerves are 200-fold more sensitive to nicotine than are TRP channels. Of note, nicotine (20 mM)-induced CGRP release was more pronounced at alkaline pH, allowing for the deprotonated weak base nicotine to freely pass the plasma membrane,^29^ which is consistent with the intracellular binding site of the TRP channels.^20^ These high millimolar nicotine concentrations may indeed be reached in the oral cavity when using NRT products,^29^ underlining the relevance of TRP channels in the irritating effect induced by nicotine. In the mouse buccal mucosa, the nAChR-mediated activation of CGRP release induced by concentrations of up to 20 mM nicotine as found in the trachea was missing—probably due to the hydrophobic diffusion barrier. However, when applying 20 mM nicotine at alkaline pH, a robust response mainly mediated by TRPA1 and TRPV1 channels was achieved.^30^ Relevant irritant effects of spices, cannabis, cigarettes, and nicotine on TRP and nAChR channels expressed in the upper airways and oral cavity are listed in Supplementary Table 1 with the irritants agonistic/antagonistic actions and an estimation of their target receptor binding potential.

## TRP Channels as Attractive Targets to Modulate Nicotine-Elicited Oral Irritation and Pain

The TRP superfamily of non-selective cation channels is composed of polymodal transducers that can be activated by mechanical, osmotic, thermal and chemical stimuli.^31^ Their role in nociceptive transduction and pain is well established,^32^ together with many other clinical applications.^33^

**Figure 2.**
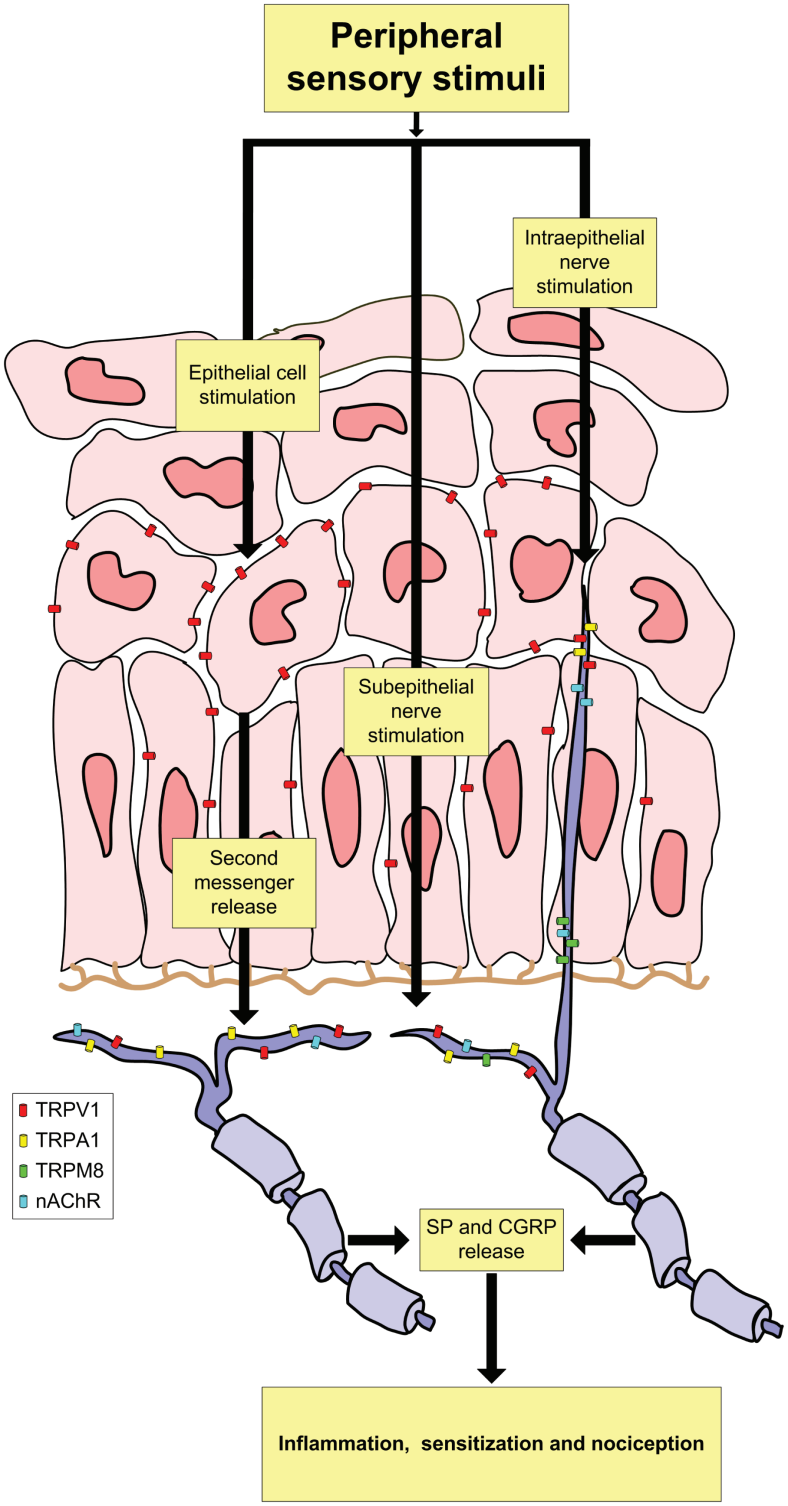
Schematic representation of TRP and nAChR receptors in the oropharyngeal mucosa and their mechanism of action. TRPV1 = transient receptor potential vanilloid 1; TRPA1 = transient receptor potential ankyrin 1; TRPM8 = transient receptor potential melastatin 8; nAChR = nicotinic acetylcholine receptor; SP = substance P; CGRP = calcitonin gene-related peptide.

The potentially relevant TRP channels for the interaction with nicotine’s oral irritating action mainly comprise TRPM8, TRPA1, and TRPV1. Expression of these receptors by nociceptive nerve fibers in TGs^34^ underlines their importance in transduction of nociceptive stimuli and pain. TRPM8, TRPA1, and TRPV1 are widely expressed throughout the human oropharynx and larynx: a quantitative RT-PCR and immunohistochemical analysis revealed localization of TRPV1 in epithelial cells and sensory fibers in the oropharyngeal submucosa with higher levels in the tongue compared to pharynx. TRPA1 was expressed below the basal lamina but not in epithelial cells.^18^ Like TRPA1, TRPM8 was localized on the sensory fibers innervating the mucosa below the basal lamina throughout the oropharynx and larynx (Figure 2).^19^

The vanilloid (capsaicin) receptor TRPV1 is a channel sensitive to noxious heat, as well as to pH decrease, endovanilloids, and pungent plant compounds including capsaicin, camphor, and eugenol.^31,35^ TRPV1-mediated stimulation of nociceptive neurons is perceived as a burning sensation. The initial pain sensation is followed by a lasting refractory state, called desensitization,^36^ a principle that is therapeutically used with topical capsaicin treatment in postherpetic neuralgia. Furthermore, pro-inflammatory agents such as serotonin, bradykinin or tumor necrosis factor alpha can sensitize TRPV1 to activation by agonists, for example, capsaicin. Thus, TRPV1 functions as a molecular amplifier of painful stimuli in sensory neurons.^35^

TRPA1 is activated by noxious cold, heat, and mechanical stimuli^37^ and by pungent and irritant compounds containing highly reactive electrophilic carbon atoms including allyl isothiocyanate (mustard oil), allicin (in garlic), formaldehyde, and tear gas components.^38^ TRPA1 is largely co-localized with TRPV1 on peptidergic nociceptive neurons in TGs and DRGs producing neuropeptides including CGRP, substance P and neurokinin A^39^ in rodents and humans^40^ and is implicated in inflammatory pain.^39^ Furthermore, in a human psychophysical study, inhalation of cigarette smoke containing a high concentration of nicotine induced a sensation of irritation and coughing.^41^ Using human bronchial and alveolar epithelial cell lines, cigarette smoke-induced cell damage was largely reduced after pharmacological inhibition or gene knockout of TRPV1 and TRPA1, corroborating the role of both channels in inflammatory conditions.^42^ Thus, TRPA1 is believed to be a key player in pain and inflammation, and pharmacological antagonists may be used as analgesic drugs.

The cold and menthol receptor TRPM8 is a low-threshold cold-sensing receptor and represents a well-established molecular pain target, but it is often debated whether TRPM8 activation is pro-algesic or analgesic.^31^ In transgenic mice, among all TRPM8-positive TG neurons, 26% stained for CGRP and among them 93% also for SP. These neurons projected predominantly through the mandibular nerve to intraoral structures.^43^ Among the sensory ganglia, TG have the highest number of TRPM8-positive neurons. TRPM8 was shown to be co-localized with the nociceptive marker TRPV1 in about 10%–40% of TG neurons in mice, underlining its potential role in pain transduction. TRPM8 is activated by cooling agents such as menthol and icilin.^31^ If menthol (2 mM) is combined with cooling (14°C), CGRP is released from mouse buccal mucosa in a TRPM8-dependent manner.^30^ However, menthol is a promiscuous compound since it does not selectively activate TRPM8 but also interacts with additional molecular targets involved in pain transduction (see below).

## Natural Agents and Their Putative Interaction with the Irritating Effect of Nicotine—Menthol, Other Monoterpenes, and Pungent Compounds

Since the cyclic monoterpene alcohol menthol, a nonselective TRPM8 agonist, is the most common flavor additive to cigarettes,^44^ many studies have been conducted to unravel its physiologic and pharmacologic actions, especially in regard to its interaction with nicotine. Natural mint oil contains ~98% (−)-menthol that carries the minty smell associated with peppermint and that has a lower sensory detection threshold for cooling than (+)-menthol.^45^ The cooling effect of menthol on the skin and mucosa is mediated by the cold-sensitive ion channel TRPM8 that is expressed by sensory nerve fibers.^46^ The analgesic effect of menthol found in animal models is thought to be mainly mediated by TRPM8 as well, as has been demonstrated using different genetic and pharmacological approaches in mice.^47^ Whereas low concentrations of menthol applied to skin or mucosa cause a cooling sensation, higher concentrations are also irritating and cause a burning pain.^45^ Recently, Lin et al. suggested that menthol in cigarettes also contributes to lung inflammation associated with smoking: the inflammatory response assessed in a mouse model of subchronic cigarette smoke exposure was mediated by TRPM8 expressed in lung epithelial cells.^48^

Besides its effect at TRPM8, menthol also interacts with other molecular targets involved in pain signaling. Menthol concentrations in the middle to high micromolar range were shown to modulate Cys-loop ligand-gated ion channels by interaction via allosteric binding sites: positive modulation of inhibitory gamma aminobutyric acid (GABA)A and glycine receptors was demonstrated for (+)-menthol.^49^ Menthol also stereoselectively inhibited excitatory 5-hydroxytryptamine type 3 (5-HT_3_) receptors.^50^ The observed stereoselective effect of menthol in these studies confirms a specific interaction with ligand-gated ion channels and argues against a nonspecific effect on membrane fluidity. Most notably, menthol was shown to be a negative allosteric modulator of native nAChRs of rat TGs and heterologously expressed human a4β2 nAChRs^51^ and α7 nAChRs. Furthermore, menthol enhanced the desensitization of heterologously expressed human a3β4 nAChRs.

In the mouse trachea, menthol (1 µM–10 mM) had no effect on (−)-nicotine (100 µM)-evoked CGRP release which depends exclusively on nAChR activation (Kichko and Reeh, 2008, unpublished observations).

In addition, high micromolar concentrations of menthol inhibited neuronal voltage-dependent Ca^2+^ and Na^+^ channels.^52^ The interaction with voltage-dependent Na^+ ^channels may explain the known local anesthetic properties of topically applied menthol.^53^

From the TRP channel family, TRPA1 is another target for menthol. In studies with mouse TRPA1 channels, menthol exhibited a bimodal effect with activation at submicromolar to low micromolar concentrations and inhibition at higher concentrations >100 µM.^54^ Consistently, menthol was able to desensitize nicotine-induced activation of heterologously expressed mouse TRPA1 channels and furthermore reduced the TRPA1-mediated airway constrictive effect evoked by nicotine in mice.^26^ In contrast, in another study with the human TRPA1 channel menthol did not inhibit but only activated TRPA1.^55^ Thus, caution must be taken when extrapolating menthol’s effects from animals to humans. Furthermore, menthol also acts as an activator of the innocuous heat receptor TRPV3.^56^

In the mouse buccal mucosa, menthol (10–20 mM) had no effect on nicotine (20 mM, pH 9) -evoked CGRP release which depends on TRPA1/V1 activation.^30^

In regard to the sensory interaction between menthol and nicotine, it was demonstrated early on that menthol cross-desensitized nicotine-evoked oral irritation in humans (see also below).^57^

It is not surprising that structurally similar naturally occurring compounds from the monoterpene class show target receptor profiles comparable to menthol and provide promising strategies to counteract the local irritation elicited by nicotine.

Similar to menthol, the bicyclic monoterpene camphor is topically applied for its antipruritic, analgesic, and counterirritant properties.^58^ Its ability to modulate warmth sensations has been attributed to its activation of the innocuous heat receptor TRPV3.^59^ As has been found for menthol,^54^ camphor exhibited a bimodal action on mouse TRPA1 with channel activation at concentrations <300 µM and inhibition at higher concentrations.^60^ Furthermore, low millimolar concentrations of camphor activated human heterologously expressed and native rat TRPM8 and sensitized the receptor to cold temperatures.^61^ In a study with bovine adrenal chromaffin cells, camphor in the mid micromolar concentration range also non-competitively inhibited nAChRs,^62^ as has been reported for menthol.^51^ The specific inhibitory effect of camphor on nAChRs was confirmed in a study measuring the nicotine-stimulated release of CGRP in the mouse trachea as well as in cultured sensory neurons.^20^

Eucalyptol (1,8-cineol), a main constituent of eucalyptus oil, was shown to inhibit heterologously expressed human TRPA1 and to activate heterologously expressed human TRPM8.^63^ Mouse models revealed anti-inflammatory properties of 1,8-cineol that were mediated by TRPM8.^64^ Notably, 1,8-cineol also blocked voltage-gated Ca^2+^ channels in tracheal smooth muscle thereby mediating a relaxation effect.^65^

Additional relevant bicyclic monoterpenes exhibit an even more potent inhibition of heterologously expressed human TRPA1 channels compared to camphor (half-maximal inhibitory concentration [IC_50_] 1.26 mM) and 1,8-cineole (IC_50_ 3.43 mM). These include borneol (IC_50_ 0.2 mM), 2-methylisoborneol (IC_50_ 0.12 mM), and fenchyl alcohol (IC_50_ 0.32 mM).^66^ In particular, borneol—established as a traditional Eastern medicine—has shown antinociceptive and anti-inflammatory activity in animal studies.^67^ In Ca^2+^ imaging experiments, borneol also inhibited nicotine-induced responses in subpopulations of mouse TG neurons.^68^ Similar to camphor and menthol, concentrations of borneol in the mid micromolar range non-competitively inhibited nAChRs in bovine adrenal chromaffin cells.^69^ Based on these results, inclusion of borneol or better isoborneol, due to its preferable smell and greater capacity to inhibit TRPA1/V1 in the oral cavity (Kichko and Reeh, unpublished data), may prove useful in oral NRT products.

Another potential strategy to alleviate nicotine-induced irritation is the co-application of pungent natural products such as curcumin or mustard oil. Micromolar concentrations of curcumin were shown to activate and subsequently desensitize heterologously expressed human TRPA1 as well as native TRPA1 of mouse sensory neurons.^70^ Similar results were obtained in vivo with the synthetic TRPA1 agonist capsazepine, which was originally the first TRPV1 antagonist. In mice, capsazepine administered in the drinking water induced a body-wide desensitization against noxious heat, capsaicin, and the prototypical TRPA1 agonist mustard oil delivered to the eyes, colon and skin.^71^

In conclusion, naturally occurring monoterpenes represent promising agents to alleviate the local irritation elicited by nicotine via interaction with a multitude of relevant molecular targets. The interference of these compounds with TRPA1 function may limit pathological nociception. Likewise, functional desensitization of TRPA1 by applying pungent natural agents may counteract the oral pain sensation elicited by nicotine.

Menthol and presumably other structurally related monoterpenes are rapidly absorbed after oral administration, mainly in the upper parts of the small intestines, and thus are systemically available. These small, lipophilic compounds are assumed to penetrate through the blood-brain barrier. Menthol and borneol are even used to enhance penetration of the blood-brain barrier by other drugs such as antitumor agents.

## Psychophysical and Neurobiological Studies of Oral Irritation—Sensitization, Desensitization, and Cross-Desensitization Effects

Human psychophysical studies to track the intensity and time course of oral irritation have been performed using capsaicin and piperine. Repeated applications of these pungent compounds to the tongue led to increased intensities of irritation, a process termed `sensitization’. The sensitization may result from an increased sensitivity of peripheral receptors (`peripheral sensitization’) or of trigeminal spinal subnucleus caudalis neurons (‘central sensitization’). However, following a rest period, reapplication of capsaicin elicited much less irritation, which is termed ‘self-desensitization’ that may be explained by peripheral or central depression of trigeminal activity as confirmed in neurobiological studies.^72^

In a bioassay performed in human PC-3 cells, which express the TRPV1 receptor, capsaicin and piperine had similar pharmacodynamics showing the maximum activation peak with a concentration of 10^−5^ M and 10^−3^ M, respectively. These TRPV1 agonists showed significant desensitization after a second application, and their effects were inhibited by a TRPV1-specific antagonist (SB366791).^73^

In contrast to capsaicin-induced sensitization, the irritation elicited by nicotine declines in intensity across repeated applications to the human oral mucosa,^74^ presumably resulting from tachyphylaxis of nAChRs to repeated application of nicotine. The observed self-desensitization after application of 300 mM (10 µL) nicotine to the tongue lasted for at least 24 h, indicating that oral intake of high nicotine amounts, for example, contained in NRT products, reduces the irritative sensation of subsequent applications.^75^ Similar patterns of self-desensitization were also found for other compounds such as menthol and mustard oil.^72^

Nicotine-elicited irritation was less pronounced after pretreatment with capsaicin, a process termed ‘cross-desensitization’. However, cross-desensitization of the capsaicin response was absent after pretreatment with nicotine,^74^ but a high dose of oral nicotine (300 mM) cross-desensitized the irritation evoked by capsaicin.^76^

## Evidence on the Interaction Between Nicotine and Natural Compounds in the Oral Cavity from Human Studies

Dessirier et al. investigated the interaction between nicotine and menthol in a psychophysical study. Preapplication of menthol (19.2 mM) to the tongue diminished the perceived intensity of irritation elicited by subsequent application of nicotine (37 mM), consistent with cross-desensitization. Menthol’s counterirritant effect was independent from its cooling ability, as the reduced intensity of nicotine-induced irritation was still present after the cooling effect had dissipated.^57^ Most cross-desensitization human studies cannot exclude that the central descending modulatory pathways (i.e. conditioning pain modulation) may interact with the results and to some degree mimic cross-desensitization; for example, itch is inhibited by a conditioning pain stimulus.^77^

In another study, chemosensory stimuli (nicotine, menthol) were nasally applied using an olfactometer. In accordance with previous results,^57^ stinging and burning sensations elicited by brief (1 sec) repeated application of nicotine initially increased followed by a slow decrease, consistent with self-desensitization. In contrast to the findings of Dessirier et al.,^57^ tonic menthol application (5.1–21.7 µM), introduced after the 15th nicotine stimulus, did not influence pain ratings for nicotine (0.6 and 0.8 µM). Whereas the lowest applied menthol concentration of 5.1 µM was just above the olfactory detection threshold, the highest concentration of 21.7 µM was associated with a cooling effect and a “cutting” pain sensation.^78^ Possible explanations for the contradictory results are (1) much lower concentrations of menthol and nicotine applied in the latter study compared to the Dessirier study, or (2) that trigeminal nerve endings are closer to the surface in the ocular and nasal mucosa and thus more sensitive to airborne chemicals, compared to oral mucosa. In the oral mucosa, trigeminal endings are located deeper in the lingual epithelium and form paragemmal baskets around the basal part of taste buds and possibly penetrate into the lumen of the taste bud-intragemmal.

Concentration-dependent interaction of nicotine and menthol in the oral cavity was also demonstrated in a study involving smokers, in which different concentrations of nicotine (6–24 mg/mL) and menthol (0.5%, 3.5%) were inhaled via E-cigarettes. A low concentration of menthol (0.5%) did not affect perceived irritation and harshness elicited by nicotine. In contrast, a higher menthol concentration (3.5%) combined with a low nicotine concentration (<18 mg/mL) resulted in increased perception of irritation compared to nicotine alone, while the high menthol concentration (3.5%) attenuated the irritation elicited by the highest nicotine concentration (24 mg/mL). These effects could be mediated by a peripheral or central mechanism. The absence of menthol’s analgesic action at low nicotine concentrations may be explained by menthol’s ability to produce irritation at high concentrations that was probably stronger than the irritation produced by low nicotine concentrations.^79^

Further evidence for the complex sensory profile of nicotine came from a study where menthol was added to nicotine gums of different concentrations (2 mg, 4 mg).^80^

Co-application of menthol transiently reduced the irritation/pain intensity but not the area of nicotine-induced irritation within the first 4 min of chewing, supporting the cross-desensitization reported in earlier studies. However, an animal study^47^ showed that TRPM8 was the principal mediator of menthol-induced analgesia via an opioid-dependent analgesic pathway, which likewise may contribute to the above data.^80^

It was found that the taste of the nicotine and menthol combination was bitter^80^ as both nicotine and menthol have a bitter taste. However, the only study looking at effects of tastants on capsaicin-evoked irritation concluded that quinine, a bitter tastant, was relatively ineffective in reducing capsaicin irritation.^81^

Nicotine-containing gums often cause irritation of several areas: on the sides of the tongue, hard palate, and throat. Although perceived pain and irritation intensities as well as the sizes of irritation areas were similar between 2 mg and 4 mg nicotine, the 4 mg dose resulted in continued increases in the intensity and area of irritation in the throat post-chewing.^80^ Of note, 12 of 22 volunteers responded to menthol as an irritant and these individuals responded more strongly the irritation of nicotine and larger areas of irritation in the throat.^80^ The observed irritant-responder profile may be explained by functional differences in pharmacological targets such as TRPA1. These findings underscore the challenge for reducing the nicotine-induced oral irritation.

In a subsequent study applying the same design, the interaction of nicotine (4 mg) and cinnamaldehyde (20 mg)—an irritant predominantly activating TRPA1^82^—was assessed in non-smokers.^83^ Both compounds elicited irritation and pain in the oral cavity. However, cinnamaldehyde added to nicotine did not affect the intensity of nicotine-induced intraoral burning or irritation.

These differences may influence the desensitizing power of compounds although this was not studied in detail. As has been demonstrated for menthol,^80^ half of the participants responded to cinnamaldehyde as an irritant, and these responders also reported larger nicotine-induced pain/irritation areas,^83^ corroborating the existence of an irritant-responder profile based on the molecular structure of pharmacological targets. The question remains if adding cinnamaldehyde to nicotine gum for smoking cessation may be beneficial or not.

Potential differences between smokers and non-smokers in intraoral somatosensory, vasomotor and temperature responses to the TRPA1 agonists nicotine (4 mg), menthol (30 mg) and cinnamaldehyde (25 mg) delivered via chewing gums have been investigated.^84^ The increases in temperature, heart rate, and blood pressure in response to the 10-min chewing regimen of menthol, nicotine, and cinnamaldehyde gums were similar between smokers and non-smokers. In general, long-term exposure to nicotine did not influence the intensities or peak responses of burning, cooling and irritation. Nicotine burn was predominantly located at the back of the throat and cinnamaldehyde burn on the tongue with no differences between smokers and non-smokers. In contrast, the cooling sensation upon menthol application was more widely distributed in the mouth of non-smokers as compared to smokers, which may have an impact on menthol’s counterirritant and/or cross-desensitizing properties in NRT products. Remarkably, smokers demonstrated a larger area of cinnamaldehyde-elicited irritation in the throat. Whereas the frequencies of reported burning upon application of menthol or cinnamaldehyde did not differ between smokers and non-smokers, burning in response to nicotine was more often reported by smokers (92%) than non-smokers (63%). Thus, smoking individuals may be more likely to show an irritation response to potentially noxious substances. Nevertheless, the dynamic response to TRPA1 agonists appears to be intact in smokers, which is important for the design of effective oral NRT products.

## Clinical Effect of TRPV1, TRPA1, and TRPM8 Agonists on the Swallowing Response

Activation of TRP channels improves both the biomechanics and the neurophysiology of the swallowing function.

In a series of comparative studies on the relative potency of TRP agonists to stimulate the swallow response in patients with oropharyngeal dysphagia, the supplementation of boluses with the TRPV1 agonist capsaicin (10 or 150 µM) had the most favorable therapeutic effect on videofluoroscopic signs and swallow response compared with the dual TRPV1/TRPA1 agonist piperine (150 µM or 1 mM), the TRPA1 agonist cinnamaldehyde (100 ppm), and the TRPM8 agonist menthol (TRPV1 > TRPA1 > TRPM8).^85^ This improvement in the oropharyngeal swallow response resulted in a significant improvement in safety of swallowing in these patients.^86,87^ Regarding the neurophysiological response, some agonists like capsaicin and cinnamaldehyde induced a shortening of the latency and an increased amplitude of the characteristic peaks in the pharyngeal sensory-evoked potential, resulting in an increased conduction and integration of sensory input and increased cortical activation.

Hence, TRP agonists are promising pharmacologic therapeutic strategies for patients with oropharyngeal dysphagia.^86^

## Role of nAChRs in the Esophageal Responses (Striated and Smooth)

NAChRs are major players in oropharyngeal and esophageal function as all muscles involved in the pharyngeal phase of swallowing are striated muscles use acetylcholine as their neurotransmitter. The innervating motor nerves are myelinated, contain choline acetyltransferase and CGRP and nAChRs cause muscular contraction. In vitro animal studies have clearly shown that stimulation of nAChRs in the oropharynx causes longitudinal and circular contraction.^88^

In contrast, the distal two-thirds of the human esophagus are composed of smooth muscle cells innervated by excitatory enteric motor neurons that release ACh activating muscarinic ACh receptors, as well as by inhibitory enteric motor neurons releasing nitric oxide.

In organ bath studies using human tissue, enteric motoneurons tonically inhibit lower esophageal sphincter myogenic tone and are effectively activated by nAChRs located in the somatodendritic regions and nerve terminals, causing strong relaxation of the lower esophageal sphincter.^89^ In contrast, although esophageal excitatory enteric motor neurons are efficiently stimulated by electrical field stimulation, their stimulation through nAChRs only elicits weak responses.^89^

## Stimulation of Saliva Production via nAChRs AND TRP Activation and the Impact on NRT

The parotid, submandibular, and sublingual salivary glands secrete saliva in response to molecular sour, sweet, salt, bitter and umami stimulation of taste receptor cells (TRCs), which are concentrated on the dorsal surface of the tongue.^90^

Different taste receptors are sensitive to sweet/ umami, bitter, sour, and salt and through intracellular calcium signaling and/ or membrane depolarization release neurotransmitters that activate primary afferent fibers supplying TRCs and reflex activation of salivary secretion.^91^

Of these stimuli, sour tastants evoke a copious secretion of salivary fluid, while sweet and bitter tastants evoke lesser fluid secretion but can alter the protein composition of saliva.^92^ A study using nicotine-containing chewing gum concluded that the addition of nicotine did not enhance salivary secretion above that of the chewing gum base.^93^ However, bitter tastants including nicotine have been shown to activate bitter receptors (TAS2Rs)^94^ co-expressed by TRCs. Therefore, it is likely that small amounts of saliva secretion, as well as altered salivary protein secretion and composition are evoked by nicotine acting at TAS2R. Besides its elicited taste responses through peripheral TRPM5-dependent pathways, common to other bitter tastants, nicotine can also activate gustatory neurons in the chorda tympani by a second TRPM5-independent pathway through nAChRs expressed on TRCs and gustatory neurons.^95^

It is unclear how stimuli acting via nAChRs in gustatory neurons might modify bitter tastant-evoked secretion of saliva.

Intravenous injection of nicotine evokes increased secretion of salivary fluid.^96^ In ex vivo preparations of parotid gland secretory tissue, micromolar concentrations of nicotine increased intracellular Ca^2+^, but the response was dependent upon the presence of autonomic nerve terminals attached to the acinar cell units. The nicotine-evoked Ca^2+^ response was largely blocked by a muscarinic AChR antagonist and partly blocked by an α1-adrenergic receptor antagonist. The response was also blocked by mecamylamine, a relatively selective nAChR α3β4 antagonist, suggesting that salivary secretion induced by nicotine is due to release of acetylcholine and noradrenaline from autonomic nerve terminals through activation of the α3β4 nicotinic receptor subtype.^96^

Major salivary glands and minor salivary glands beneath the oral epithelium secrete saliva in response to chemosensory stimulation of trigeminal nerve endings by capsaicin, nonivamide and other TRPV1 agonists.^97^ Menthol, a TRPM8 agonist, can also increase saliva secretion and modulate the protein composition of saliva, but cinnamaldehyde, a TRPA1 agonist, had relatively little effect on salivation.^97^ Nicotine-mediated salivary secretion might therefore be evoked through activation of nAChRs expressed by trigeminal nerve endings in the oral cavity and at higher nicotine concentrations through activation of TRPV1 channels expressed in sensory nerves of the trachea.^30^

A major function of saliva secreted into the oral cavity is clearance of food and other ingested exogenous materials. This would be expected to modify the absorption of nicotine through oral epithelial surfaces and the perception of bitterness/pungency of nicotine. In the absence of salivary secretion elicited by taste or chewing, the half-life for oral clearance of nicotine, if similar to other studied small molecules (e.g. sucrose and fluoride), can be estimated as 2 min, given a mean flow rate of 0.3 mL/min.^98^ If oral nicotine is delivered in a chewing gum vehicle the clearance rate will be considerably faster. The pH of saliva secreted in response to chewing is approximately 7.6 compared to approximately 6.6 for baseline salivary pH, a difference that will alter the proportion of unionized nicotine and potentially the amounts absorbed through oral mucosal surfaces since saliva forms a thin, mobile film on the oral epithelium.^99^ A final consideration is that nicotine is likely to interact with components of saliva, which may alter the perception and absorption of nicotine in the oral mucosa. For example, spectroscopic studies suggest that nicotine can interact with the aromatic amino acids of salivary mucin, an important determinant of the salivary film on the oral epithelium.

## Future Perspectives

Nicotine is the main irritant in cigarette smoke; the smoker has learned to mix the smoke with air to an extent that allows inhalation without coughing and distress. A successful oral NRT should most likely contain some volatile irritant to be released while chewing. Ideally, in addition, this compound should either desensitize or inhibit the TRPA1/V1 receptors in subepithelial oral nociceptors or induce reflex inhibition of nociceptive transmission in the trigeminal nuclear complex.

Cross-desensitization of nicotine irritation by menthol at high concentrations might activate TRPA1 to induce some burning pain. However, much lower concentrations of menthol are required to activate TRPM8 in the A-delta low-threshold cold fibers in humans. These particular fibers provide segmental input to a spinal dorsal horn circuitry that inhibits nociceptive transmission; this mechanism is probably working also in the trigeminal system. Such a spinally-mediated inhibition through TRPM8 has previously been suggested.^100^

As referred to in the review, many studies are conducted to assess the irritating properties of nicotine-containing products in the oral cavity and the potential counterirritant effect of naturally occurring compounds that can be safely used in humans, However, most research data stem from studies conducted in animal or cell models. Since differences between humans and rodents exist the data obtained from animal studies cannot be directly extrapolated to humans without restrictions. As pointed out, the available results for menthol’s counterirritant properties from human studies appear to be well in accordance with the data from cell and animal studies.

It is known that TRPV1, TRPA1, and TRPM8 are widely expressed in the human oropharynx and larynx with distinct patterns of expression/location. As mentioned, this has led to pharmacologic studies using capsaicinoids, piperine, cinnamaldehyde, and menthol to modulate swallow responses. In addition, nAChRs are major players in oropharyngeal and esophageal function causing contraction of striated muscles. In the lower esophageal sphincter, they cause stimulation of non-adrenergic, non-cholinergic myenteric inhibitory motor neurons and lower esophageal sphincter relaxation.

A promising strategy to counteract the nicotine-induced side effects to some degree, and thereby potentially increasing adherence to NRT, may be the addition of natural GRAS compounds such as monoterpenes to oral NRT products. As mentioned, these compounds have been demonstrated to interact with a broad spectrum of molecular targets involved in pain signaling, most importantly with TRP channels and nAChRs.

In summary, human studies are warranted to test the potential masking effects of promising natural compounds such as those with TRPA1-antagonistic properties. Such masking effects can be peripherally or centrally mediated and the role of those two sites of action and the possible utilization of both are currently not known and should be addressed in future studies.

## Supplementary Material

A Contributorship Form detailing each author’s specific involvement with this content, as well as any supplementary data, are available online at https://academic.oup.com/ntr.

ntac054_suppl_Supplementary_Material

ntac054_suppl_Supplementary_Taxonomy-form
