# Supplementary material for: The Role of TRP Channels in Nicotinic Provoked Pain and Irritation from the Oral Cavity and Throat: Translating Animal Data to Humans
Source: Nicotine Tob Res. 2022 Feb 24;24(12):1849–60. doi: 10.1093/ntr/ntac054 (PMC9653082; doi:10.1093/ntr/ntac054)
Supplement: ntac054_suppl_Supplementary_Material [file ntac054_suppl_supplementary_material.docx]

**Supplementary table 1. Irritant effects of spices, cannabis, and nicotine on TRP and nAChR channels expressed in the upper airways and oral cavity.**

|  | TRPV1 | TRPV2 | TRPV3 | TRPV4 | TRPA1 | TRPM8 | nAChR |
| --- | --- | --- | --- | --- | --- | --- | --- |
| Acidity  (pH < 7) | ++ (107) |  |  |  | +++ only hTRPA1  (112) |  |  |
| Allicin  (garlic) | ++ |  |  |  | +++ (108) |  |  |
| Allyl isothiocyanate  (mustard oil) | ++ |  |  |  | +++ (108) |  |  |
| Camphor  (camphor laurel) | + |  | ++ |  | - - (115) | ++ (114) | - - (20) |
| Cannabidiol  (cannabis sat.) | + (110) | + (110) | ++ (111) |  | + (110) | - - (109) |  |
| Cannabinol, tetrahydro  (Δ^9^−THC) | ++ (110) | ++ (110) |  |  | ++ (110) | - - (109) |  |
| Capsaicin  (chili peppers) | +++ (104, 108) |  |  |  |  |  |  |
| Cardamom  (1,8-cineol) |  |  |  |  | ++/- - | ++ (104) |  |
| Carvacrol  (oregano) |  |  | ++ |  | +++ (106, 108) |  |  |
| Cigarette smoke  (gas phase) |  |  |  |  | +++ (14) |  |  |
| Cigarette smoke  (full smoke) |  |  |  |  | +++ (14) |  | + (14) |
| Cinnamaldehyde  (cinnamomum trees) |  |  |  |  | +++ (108) |  |  |
| Citral  (lemongrass) | ++ | - - | ++ | - - | ++ | ++/- - (113) |  |
| Cuminal  (cumin) |  |  |  |  | ++ (104) |  |  |
| Curcumin  (curcuma) | - - |  |  |  | ++/- - (70) |  |  |
| Eucalyptol  (1,8-cineol) |  |  |  |  | - - | ++ (63) |  |
| Eugenol  (clove) (102) | ++ |  |  |  | ++ (108) |  |  |
| Galganal  (galganal root) |  |  |  |  | +++ (108) |  |  |
| Gingerol  (Shogaols) | ++ |  |  |  | ++ (108) |  |  |
| Isoborneol  (rosemary, sage) |  |  |  |  | - - (66) |  |  |
| Limonene  (lemon, orange, pine) |  |  |  |  | +/- - - (105) |  |  |
| Linalool  (coriander) |  |  |  |  | ++ (104) |  |  |
| Menthol  (mint) |  |  |  |  | ++ hTRPA1 | +++ (104, 108) |  |
| Nicotine  (tobacco) (103) | + |  |  |  | ++ |  | +++ (20, 30) |
| Piperine  (black pepper) | ++ |  |  |  | ++ (104, 108) |  |  |
| Sanshool, hydroxyl α−  (Szechuan pepper) (101) | ++ |  |  |  | ++ (108) |  |  |
| Thymol  (thyme) |  |  | ++ |  | ++ (108) |  |  |

Data are compiled from diverse sources, including heterologous expression of recombinant ion channels, cultured sensory neurons, explants of vital tissues, and behavioral experiments.

**“+”** indicates agonist activity, **“-“** indicates antagonist activity, multiples of **“+”**and **“-“** are based on rough estimates of agent’s potency and effect size, **“+/-“** indicates agonism followed by desensitization. “hTRPA1” refers to human-TRPA1 in contrast to other data retrieved from animal/cell studies.

Numbers in brackets refer to the reference list in which numbers refer to extensive review articles where the original papers behind the data are cited.

References 101-115

101. Bautista DM, Sigal YM, Milstein AD, et al. Pungent agents from Szechuan peppers excite sensory neurons by inhibiting two-pore potassium channels. Nat Neurosci. 2008;11(7):772-779. doi:10.1038/nn.2143

102. Park C-K, Li HY, Yeon K-Y, et al. Eugenol inhibits sodium currents in dental afferent neurons. J Dent Res. 2006;85(10):900-904. doi:10.1177/154405910608501005

103. Gees M, Alpizar YA, Luyten T, et al. Differential effects of bitter compounds on the taste transduction channels TRPM5 and IP3 receptor type 3. Chem Senses. 2014;39(4):295-311. doi:10.1093/chemse/bjt115

104. Aloum L, Alefishat E, Shaya J, Petroianu GA. Remedia Sternutatoria over the Centuries: TRP Mediation. Molecules. 2021;26(6). doi:10.3390/molecules26061627

105. Kaimoto T, Hatakeyama Y, Takahashi K, Imagawa T, Tominaga M, Ohta T. Involvement of transient receptor potential A1 channel in algesic and analgesic actions of the organic compound limonene. Eur J Pain. 2016;20(7):1155-1165. doi:10.1002/ejp.840

106. Nazıroğlu M. A novel antagonist of TRPM2 and TRPV4 channels: Carvacrol. Metab Brain Dis. Published online January 2022:1-18. doi:10.1007/s11011-021-00887-1

107. Ni D, Lee L-Y. Effect of increasing temperature on TRPV1-mediated responses in isolated rat pulmonary sensory neurons. Am J Physiol Lung Cell Mol Physiol. 2008;294(3):L563-71. doi:10.1152/ajplung.00336.2007

108. Nilius B, Appendino G. Spices: the savory and beneficial science of pungency. Rev Physiol Biochem Pharmacol. 2013;164:1-76. doi:10.1007/112_2013_11

109. De Petrocellis L, Vellani V, Schiano-Moriello A, et al. Plant-derived cannabinoids modulate the activity of transient receptor potential channels of ankyrin type-1 and melastatin type-8. J Pharmacol Exp Ther. 2008;325(3):1007-1015. doi:10.1124/jpet.107.134809

110. De Petrocellis L, Ligresti A, Moriello AS, et al. Effects of cannabinoids and cannabinoid-enriched Cannabis extracts on TRP channels and endocannabinoid metabolic enzymes. Br J Pharmacol. 2011;163(7):1479-1494. doi:10.1111/j.1476-5381.2010.01166.x

111. De Petrocellis L, Orlando P, Moriello AS, et al. Cannabinoid actions at TRPV channels: Effects on TRPV3 and TRPV4 and their potential relevance to gastrointestinal inflammation. Acta Physiol. 2012;204(2):255-266. doi:10.1111/j.1748-1716.2011.02338.x

112. de la Roche J, Eberhardt MJ, Klinger AB, et al. The molecular basis for species-specific activation of human TRPA1 protein by protons involves poorly conserved residues within transmembrane domains 5 and 6. J Biol Chem. 2013;288(28):20280-20292. doi:10.1074/jbc.M113.479337

113. Stotz SC, Vriens J, Martyn D, Clardy J, Clapham DE. Citral sensing by Transient [corrected] receptor potential channels in dorsal root ganglion neurons. PLoS One. 2008;3(5):e2082. doi:10.1371/journal.pone.0002082

114. Vetter I, Hein A, Sattler S, et al. Amplified cold transduction in native nociceptors by M-channel inhibition. J Neurosci. 2013;33(42):16627-16641. doi:10.1523/JNEUROSCI.1473-13.2013

115. Xu H, Blair NT, Clapham DE. Camphor activates and strongly desensitizes the transient receptor potential vanilloid subtype 1 channel in a vanilloid-independent mechanism. J Neurosci. 2005;25(39):8924-8937.
